# Contextualizing Genes by Using Text-Mined Co-Occurrence Features for Cancer Gene Panel Discovery

**DOI:** 10.3389/fgene.2021.771435

**Published:** 2021-10-25

**Authors:** Hui-O Chen, Peng-Chan Lin, Chen-Ruei Liu, Chi-Shiang Wang, Jung-Hsien Chiang

**Affiliations:** ^1^ Department of Computer Science and Information Engineering, College of Electrical Engineering and Computer Science, National Cheng Kung University, Tainan, Taiwan; ^2^ Institute of Medical Informatics, National Cheng Kung University, Tainan, Taiwan; ^3^ Department of Oncology, National Cheng Kung University Hospital, College of Medicine, National Cheng Kung University, Tainan, Taiwan; ^4^ Department of Genomic Medicine, National Cheng Kung University Hospital, College of Medicine, National Cheng Kung University, Tainan, Taiwan

**Keywords:** biomedical natural language processing, machine learning, topic modeling, cancer gene panel, text mining

## Abstract

Developing a biomedical-explainable and validatable text mining pipeline can help in cancer gene panel discovery. We create a pipeline that can contextualize genes by using text-mined co-occurrence features. We apply Biomedical Natural Language Processing (BioNLP) techniques for literature mining in the cancer gene panel. A literature-derived 4,679 × 4,630 gene term-feature matrix was built. The *EGFR* L858R and T790M, and *BRAF* V600E genetic variants are important mutation term features in text mining and are frequently mutated in cancer. We validate the cancer gene panel by the mutational landscape of different cancer types. The cosine similarity of gene frequency between text mining and a statistical result from clinical sequencing data is 80.8%. In different machine learning models, the best accuracy for the prediction of two different gene panels, including MSK-IMPACT (Memorial Sloan Kettering-Integrated Mutation Profiling of Actionable Cancer Targets), and Oncomine cancer gene panel, is 0.959, and 0.989, respectively. The receiver operating characteristic (ROC) curve analysis confirmed that the neural net model has a better prediction performance (Area under the ROC curve (AUC) = 0.992). The use of text-mined co-occurrence features can contextualize each gene. We believe the approach is to evaluate several existing gene panels, and show that we can use part of the gene panel set to predict the remaining genes for cancer discovery.

## Introduction

Scientific articles provide text mining (TM) applications in cancer biology (Zhu et al., 2013; Azam et al., 2019; Wang et al., 2020). Several solutions are currently available to meet the growing need for different cancer gene panels. Several commercial gene panels constitute a “one-size-fits-all” solution. In a clinical investigation, we need to design gene panels specifically tailored for particular questions or individual cancers (Hyman et al., 2015). The precision of the designed panel for different tumors plays an important role. They rely on literature reviews and cancer genomics databases. The reason for selecting somatic and germline mutation profiling is also complicated. Emerging TM techniques such as Gene2Vec offer some answers to information interpreting problems. Gene2Vec is a study that explored the idea of gene embedding, distributed representation of genes, in the spirit of word embedding (Demeester et al., 2016; Du et al., 2019). However, we cannot explain the biomedical meaning of the vector in the neural embedding model. The goal of explainability is very important and would be very useful. The ability to provide additional gene suggestions for a gene panel with an explanation would be hugely valuable but also really challenging. Therefore, we developed a biomedical-explainable and validatable text mining pipeline for cancer gene panel discovery.

Firstly, we find a system for predicting genes and interesting applications for a gene panel discovery. The use of text-mined co-occurrences features for each gene can contextualize each gene, and as input for a machine learning system. We extract NER names mentioned in the literature, such as gene NER (Leaman et al., 2013) and disease NER (Wei et al., 2013). The use of PubTator (Wang et al., 2016) along with MeSH (Ikonomakis et al., 2005) is a good way of getting as good enrichment for biomedical relevant terms. The frequency-inverse document frequency (TF-IDF) was used to construct the document-term matrix (Ikonomakis et al., 2005). Machine learning-based and biomedical-explainable approaches have recently become the most popular approaches in the study of the document-term matrix. For example, M. Ikonomakis et al. introduced several machine learning (ML) algorithms applied to text classification such as naïve-Bayes, decision trees, neural networks, nearest neighbors, and support vector machines (Devarajan et al., 2015). Wei Xu et al. proposed a novel document-clustering method based on non-negative matrix factorization (Choo et al., 2013). Choo et al. presented a user-driven topic modeling based on interactive non-negative matrix factorization capable of tuning the topic model result by integrating user interactions (Pedregosa et al., 2011). Summarizing the abovementioned studies, we established a fully integrated text mining pipeline to find the gene term-feature, mutational landscape heatmap, and cancer information topic.

With next-generation sequencing (NGS) technologies (Shabani Azim et al., 2018), many targeted panels have been developed to detect hereditary cancer and monitor somatic mutation changes in progressive cancer (McCabe et al., 2019). The Memorial Sloan Kettering Cancer Center has developed MSK-IMPACT (Memorial Sloan Kettering-Integrated Mutation Profiling of Actionable Cancer Targets), a hybridization capture-based next-generation sequencing assay for deep target sequencing of all exons and selected introns of 410 essential cancer genes in tumors (Hyman et al., 2015; Cheng et al., 2015). The MSK-IMPACT panel performed well not only in the above study but also in a large-scale clinical sequencing project with more than 10,000 patients (Zehir et al., 2017). They provided a comprehensive gene panel database including actionable drug targets, cancer susceptibility genes in hematological malignancies, and solid tumors. For solid tumors, the Oncomine Cancer Panel (OCP) is only used for the clinical screening of actionable genetic mutations in solid tumors (Luthra et al., 2017). They significantly provide druggable target databases. We validate the biomedical literature mining through the MSK-IMPACT or OCP cancer gene panel NGS database.

We create a pipeline that can suggest additional genes for a gene panel given an existing set of genes. And we believe the approach is to evaluate several existing gene panels, and show that we can use part of the gene panel set to predict the remaining genes.

## Materials and Methods

### PUBMED

PubMed, a free database of more than 30 million literature citations for biomedical literature, includes the fields of biomedicine and health. We extracted the abstracts that mentioned genes related to human cancer from PubMed and took the gene’s context by gene window.

### Machine Learning Model and Analysis

K nearest neighbors, linear support vector machine (SVM), Gaussian process, decision tree, random forest, neural net, and naive Bayes were used to conduct supervised machine learning. All the models were built by python with the scikit-learn package and used five-fold cross-validation (Wei et al., 2015). The receiver operating characteristic (ROC) curve and the area under the ROC curve (AUC) were used to evaluate the model’s performance.

### Biomedical Term Tagging

#### PubTator

PubTator (Wei et al., 2013) is a web-based PubMed abstract biomedical named entity recognition (NER) system. PubTator can tag the gene, disease, chemical, species, and mutation in PubMed abstracts, and the tagging result could be accessed *via* the RESTful API. We used PubTator as a part of the biomedical term tagger.

#### Medical Subject Heading

MeSH is a hierarchically organized medical vocabulary thesaurus used for indexing articles for PubMed. PubMed Articles curated by NLM are indexed with several related MeSH terms; every MeSH term has unique id and hierarchical categories. With these characteristics of MeSH term and our tagging algorithm, we could tag biomedical terms that are not tagged by PubTator. Our algorithm started from the MeSH terms of each PubMed article. For each MeSH term in an article, we first created a MeSH term-mapping set that mapped a MeSH term to another set that contained itself and its lower hierarchy MeSH term. Second, for each MeSH term in the MeSH term-mapping set, we tried matching all of the entry terms, synonyms of a specific MeSH term, to every word in the article. If a word in the article matched any entry names of a MeSH term, we tagged that word as a biomedical term. This way, those terms having the same concepts could be merged and analyzed.

### Gene Term-Feature Term Frequency–Inverse Document Frequency Matrix Construction

For a particular gene, considering all of its gene windows in the whole corpus, we calculated the frequency of the co-occurrence of the gene and features (terms) tagged by our algorithm in the window as the term frequency of the feature. The higher the term frequency is, the stronger the association of the gene and feature. In our study, term frequency (TF) was calculated using the following formula:

$$TF_{gene,\;\;feature}=\text{log}\left(1+tf_{gene,feature}\right)$$

To calculate the inverse document frequency of each term feature, we simply count the occurrences of the term feature in all genes as document frequency. The inverse document frequency (IDF) was calculated using the following formula:

$$IDF_{feature}=\text{log}\left(1+n_{gene}/df_{feature}\right)$$

The higher the IDF, the more specific the term feature is to a particular gene. Finally, by multiplying TF and IDF, the gene term -feature matrix was constructed.

### Term Feature Selection by the Hypergeometric Test

We filtered out genes that had less than ten term features. We identified the critical term feature according to the gene panel using the *p*-values of hypergeometric tests as follows. We input the MSK-IMPACT (Hyman et al., 2015) panel. Ns is the size of the MSK-IMPACT panel set S, N is the size of the set $S^{'}$ , which contains 500 non-MSK genes (randomly selected from the gene term-feature matrix) and all of the MSK genes, $N_{t}$ is the number of genes in the set S' that contain term feature t, and $N_{st}$ is the number of genes in the set S containing t . The random variable y representing several genes containing the term feature in the set S is a hypergeometric random variable with parameters $N_{s},\;N_{t},\;$ and N (Westlake and Larson, 1970). The probability distribution of y is shown as follows:

$$\text{P}\left(\text{y}\right)=\frac{\left(\begin{array}{c} N_{t}\\ y \end{array}\right)\left(\begin{array}{c} N-N_{t}\\ N_{s}-y \end{array}\right)}{\left(\begin{array}{c} N\\ N_{s} \end{array}\right)}$$

From $N_{st}$ , we compute the *p*-value, the probability of the observed ( $N_{st}$ ), as follows:

$$\text{Pvalue}=\overset{\text{min}\left(N_{s},N_{t}\right)}{\underset{y=N_{st}}{\sum }}P\left(y\right)$$

The *p*-value reflects significant phrases in S compared with all of the genes in the gene term -feature matrix. A low *p*-value indicates that we observe a rare event and that the observed term feature represents a statistical discovery, suggesting that it is essential in the MSK-IMPACT panel.

### Topic Modeling

Our topic modeling was based on the algorithms of non-negative matrix factorization (NMF) (Yeganova et al., 2014). Given a nonnegative matrix $\text{X}\in {\mathbb{R}}^{m\times n}$ , when the desired lower dimension is k, the goal of NMF is to find the two matrixes, $\text{W}\in {\mathbb{R}}^{m\times k}$ and $\text{H}\in {\mathbb{R}}^{k\times n}$ , having only non-negative entries such that X≈WH .

The objective function is shown as the following formula:

$$\underset{\text{W}\geq 0,\text{H}\geq 0}{\min}f\left(W,H\right)=\left|\left|X-WH\right|\right|{}_{F}^{2}$$

The function is the most commonly used formulation based on the Frobenius norm. K represents the number of topics we expected, X represents the gene term-feature matrix, W represents the gene-topic matrix, and H represents the topic text-feature matrix. Since the weights in W and H have been calculated, we used the top 20 genes and the top 20 text features with the highest importance for each topic to interpret the biomedical meaning.

### Gene Window

We take the gene’s context as its gene window. Each gene window contains three sentences. The sentence contains the gene, the previous sentence, and the next sentence. We want to eliminate the redundant part. Using the gene window algorithm, we could iterate through the full abstracts containing specific genes in the text and grip the most critical section for further analysis. We pick three sentences based on the concept that the sentence that is closer to the gene is more relevant to it. Since the closest ones are previous and the next one, so we picked three.

## Results

### Study Design and Workflow

**FIGURE 1:**
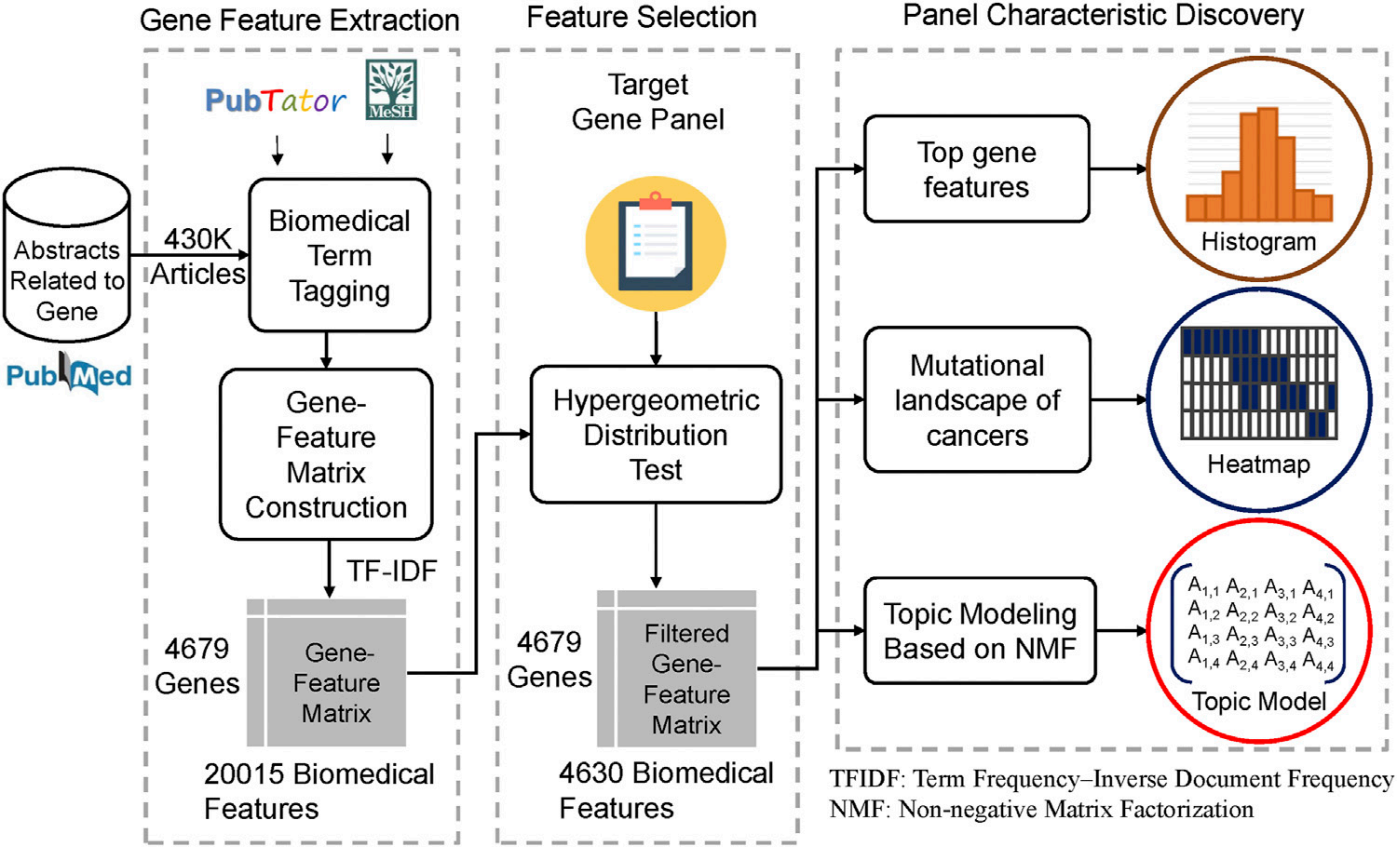
Study design and workflow The flowchart shows the overall analysis framework of this study. We first extracted 430,000 abstracts that mentioned genes related to humans in the PubMed corpus. Second, biomedical named entity recognition (NER) was performed to obtain biomedical terms, such as gene name, disease name, and drug name, using PubTator and MeSH. Third, we used the biomedical term tagged by the previous step to construct the gene term-feature matrix whose concept was similar to the document-term matrix. Fourth, we performed term feature selection according to a particular gene panel. We took the MSK-IMPACT panel as an example and made the term features generated by the previous step correspond more to the target gene panel using the hypergeometric distribution. Finally, several analyses, including identifying the top gene term features, creating the mutational landscape of cancers, and topic modeling based on nonnegative matrix factorization, were conducted to determine and interpret the biomedical characteristics of the target gene panel.

**FIGURE 2:**
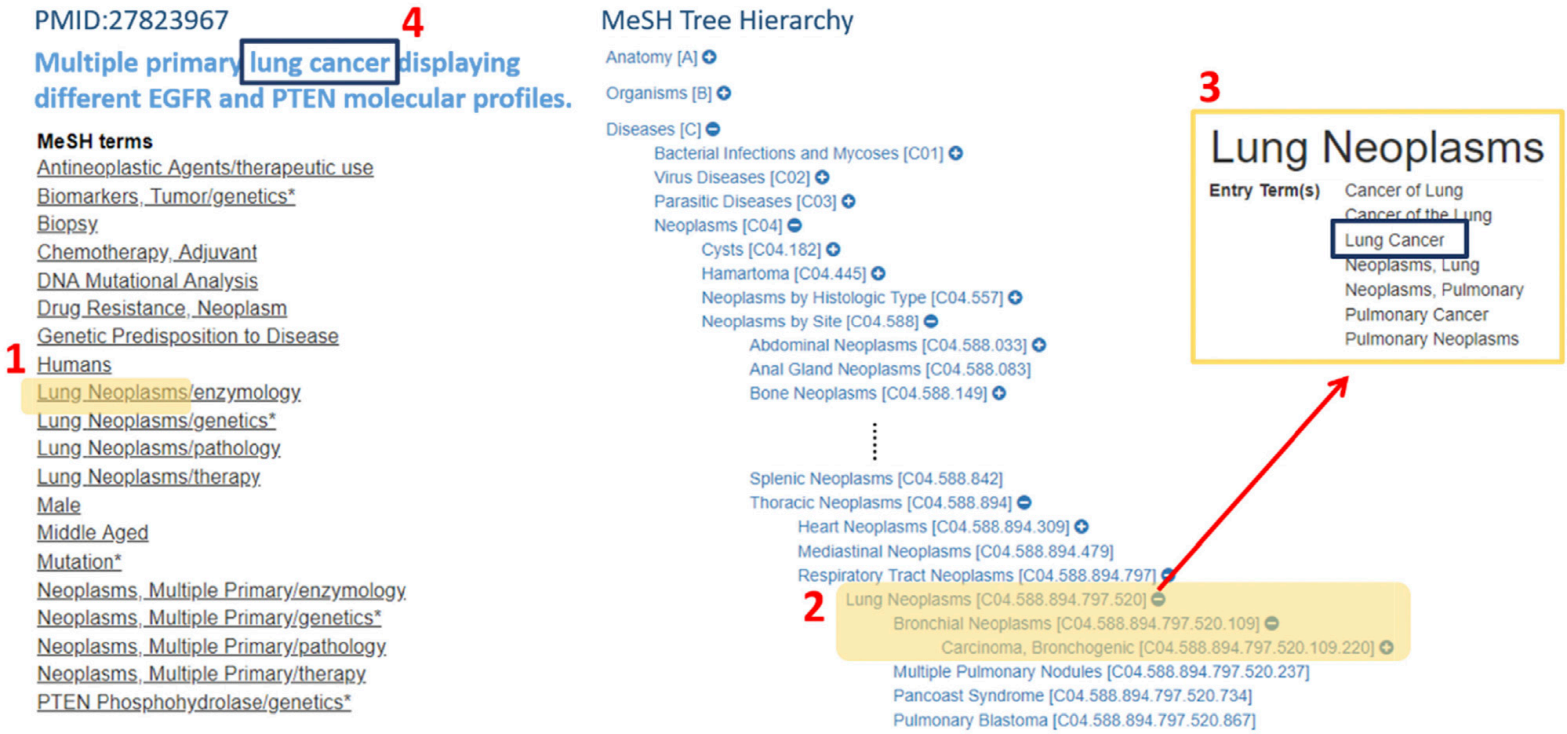
An example displays how the term “lung cancer”, being tagged in MeSH hierarchical structure. The way “lung cancer” being tagged is as follows. First, we iterate through the MeSH terms of the index of PMID: 27823967 and found “Lung Neoplasms” was one of the MeSH terms, which its synonyms contain “Lung Cancer.” Second, if the term “Lung Cancer” also appeared in the article, the MeSH tagging algorithm would tag this word and take its MeSH ID for further analysis.

This study develops a gene panel analysis framework that can discover the characteristics of a gene panel based on biomedical literature mining. The method overview is shown in Figure 1. First, we extracted the PubMed abstracts, which mentioned genes related to humans. The method is shown as Figure 2. In this step, approximately 430,000 PubMed abstracts regarding genes were filtered out from all of the current PubMed corpus (approximately 30 million articles). Second, we performed biomedical named entity recognition (NER) on the extracted PubMed abstracts using PubTator (Wang et al., 2016) and MeSH (Medical Subject Headings). Third, we used the biomedical term to construct the gene term-feature matrix, which has a concept similar to that of the document-term matrix. Fourth, we performed term feature selection according to individual gene panels to make the term feature generated by the previous step stronger and correspond to the target gene panel.

Here, we explored the idea of the hypergeometric distribution. For each term feature, by comparing the distribution of occurrences in the target gene set and the whole gene set, the term features that correlated more with the target gene panel would be enriched. This approach is flexible in regard to different target gene sets, such as the Oncomine Cancer Panel or cardiovascular gene panels. Finally, we filtered out 4,630 term features from 20,015 term features. The filtered gene term-feature matrix, whose size is 4,679 (genes) x 4,630 (term features), will be used in the following analysis. Thus, we can discover the top 20 gene term features, the mutational landscape of the cancer genome, and topic modeling of cancer information. In this way, we can find the potential characteristics of the gene panel.

### Biomedical Term Extraction by Hypergeometric Test

**FIGURE 3:**
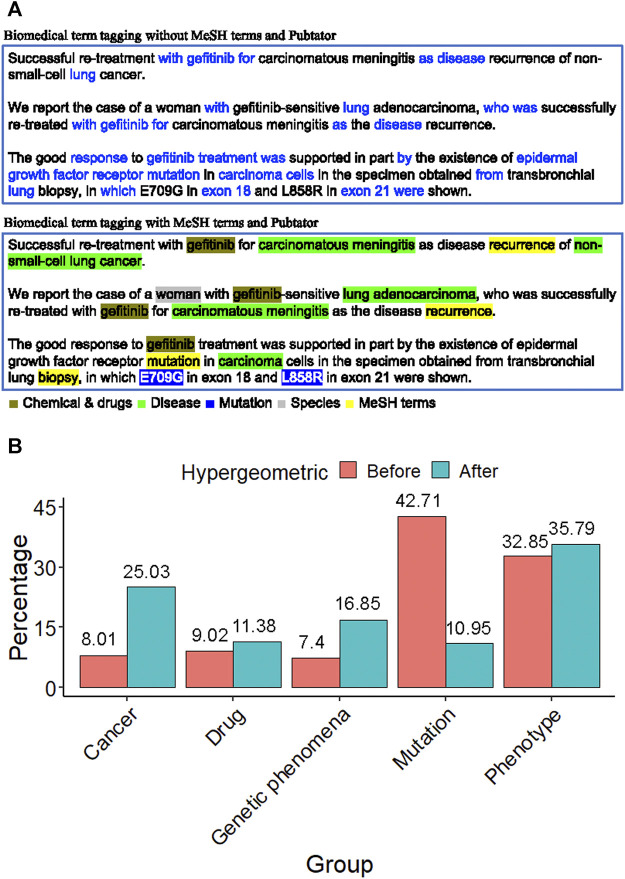
Biomedical term extraction **(A)** The term feature of an EGFR-related abstract. The former was filled with many redundant words, such as with, for, and after. The latter contains lots of biologically meaningful terms, such as gefitinib (chemical), non-small cell lung cancer (disease), L858R (mutation), the woman (species), and recurrence (MeSH). This phenomenon shows that the tagging approach with MeSH and PubTator terms is essential to gene term-feature extraction. **(B)** The proportion distribution bar chart of the MSK-IMPACT panel in each term feature group before and after the hypergeometric distribution test. It shows that after term feature selection, the proportion of the term feature groups of interest increases, such as cancer, drug, genetic phenomena, and phenotype.

In the field of biomedical literature mining, tagging the biomedical term is an important issue. For an abstract of the biomedical literature, only biomedical words are what we are interested in, such as drug name, disease name, or gene name. PubTator was capable of tagging the gene, disease, chemical, species, and mutation in PubMed abstracts. Figure 3A shows the term feature extraction result of an *EGFR*-related abstract compared to the term features extracted by raw text TF-IDF scoring without biomedical term tagging. The biomedical term features were filled with redundant words, such as “with”, “for”, and “after”.

On the other hand, the term feature extraction approach with MeSH terms and PubTator resulted in term features that contained lots of biologically meaningful terms, such as gefitinib (chemical), non-small cell lung cancer (disease), L858R (mutation), the woman (species), and recurrence (MeSH). This phenomenon shows that the tagging approach is essential for gene term feature extraction.

To discover the characteristics of a gene panel, we used the hypergeometric distribution test. According to MeSH terms and PubTator categories, all the term features can be divided into five groups: cancer, drug, genetic phenomena, mutation, and phenotype (Supplementary Table S1). Take the MSK-IMPACT panel as a target gene panel, for example. The distribution of the MSK-IMPACT panel shows that the percentage increases in some term feature groups after using the hypergeometric distribution test (Figure 3B). We filtered out the unimportant genes and found the critical term features according to the gene panel using a hypergeometric distribution test. The proportion of term feature groups in our interest increases, such as cancer, drug, genetic phenomena, and phenotype. The percentage after using the hypergeometric distribution test showed a noticeable improvement from 8.01 to 25.03% in the cancer group. The proportion increased from 9.02 to 11.38% in the drug group and grew from 7.4 to 16.85% in the genetic phenomenon group. There was a slight increase from 32.85 to 35.79% in the phenotype group. After the term feature selection, the proportion decreased from 42.71 to 10.95% in the mutation group. The MSK-IMPACT panel stands for integrated mutation profiling of actionable cancer targets, so the percentage in these groups increases after the hypergeometric distribution test.

### 3.3 Literature-derived Gene Term Features

**FIGURE 4:**
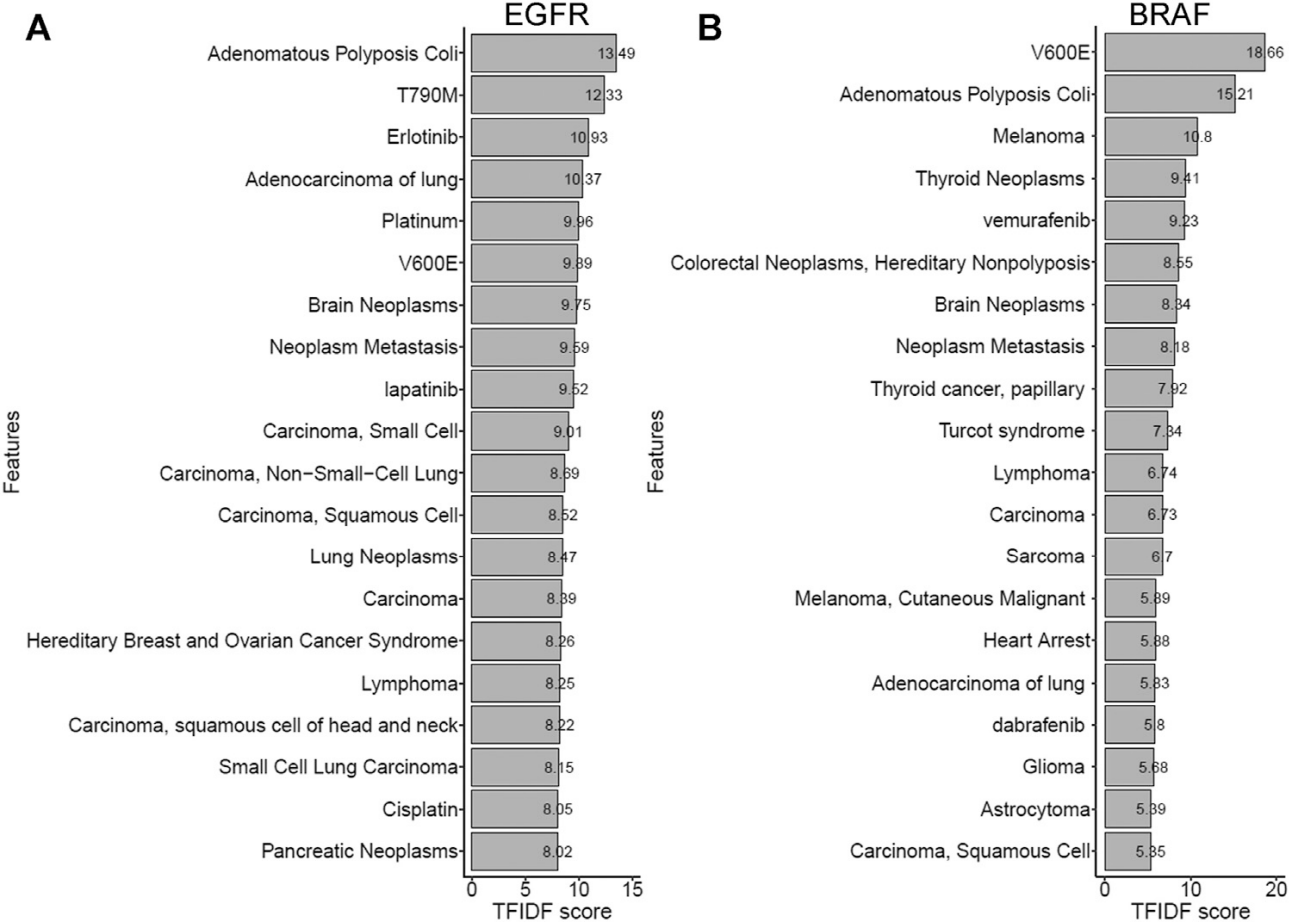
Top term features in *EGFR* and *BRAF* genes **(A)** The bar chart shows the TF-IDF scores of term features related to *EGFR*. Most of the identified term features for *EGFR* were associated with syndromes (e.g., lung adenocarcinoma and non-small cell lung carcinoma), mutations (e.g., T790M), and therapies (e.g., erlotinib and lapatinib) for lung cancer. **(B)** The bar chart shows the TF-IDF scores of term features related to *BRAF*. Biomedical term features, including cancer types (e.g., melanoma and thyroid cancer), mutations (e.g., V600E), and inhibitors (e.g., vemurafenib and dabrafenib) for *BRAF*, were consistent with known findings.

The biomedical term features extracted from the literature were directly or indirectly related to each gene. Here, we took some cancer-related genes as examples for further demonstration. Figures 4A,B show the top twenty biomedical term features with the highest TF-IDF scores for *EGFR* (the score range from 8.02 to 13.49) and *BRAF* (the score range from 5.35 to 18.66). For *EGFR*, which has been recognized for its importance in lung cancer (Paez et al., 2004; Shepherd et al., 2005), most of the term features directly represent lung cancer or its subtypes, such as “Adenocarcinoma of the lung,” “Carcinoma, small cell,” and “Carcinoma, Non-Small Cell Lung.” “T790M” is a drug resistance mutation frequently observed in patients with lung cancer (Zhou et al., 2009). “Erlotinib” is an effective tyrosine kinase inhibitor (TKI) targeting *EGFR* for non-small cell lung carcinoma (NSCLC). “Lapatinib” is a dual *EGFR/ERBB2* TKI for metastatic breast cancer (Burris, 2004). Some term features were indirectly relevant to *EGFR*, such as “Platinum” and “cisplatin,” which are both standard chemotherapy in NSCLC (Arriagada et al., 2004). *EGFR* TKIs are commonly compared with conventional platinum-based therapies. Another example is *BRAF*, whose mutations are widely detected in melanoma, thyroid cancer, and colorectal cancer (Chapman et al., 2011). “V600E” is a crucial mutation that causes the constitutive activation of the cellular signaling pathway (Chapman et al., 2011). “Vemurafenib” and “dabrafenib” are competitive inhibitors designed for *BRAF* with the V600E mutation (Hauschild et al., 2012). The other examples, such as *BRCA1*, *BRCA2*, *MLH1*, and *ERBB2*, are shown in Supplementary Figure S1. Nearly all of the biomedical term features relevant to these genes were consistent with current knowledge.

### Mutational Landscape of the Actionable Cancer Genome From Biomedical Literature Mining Validated by NGS Database

**FIGURE 5:**
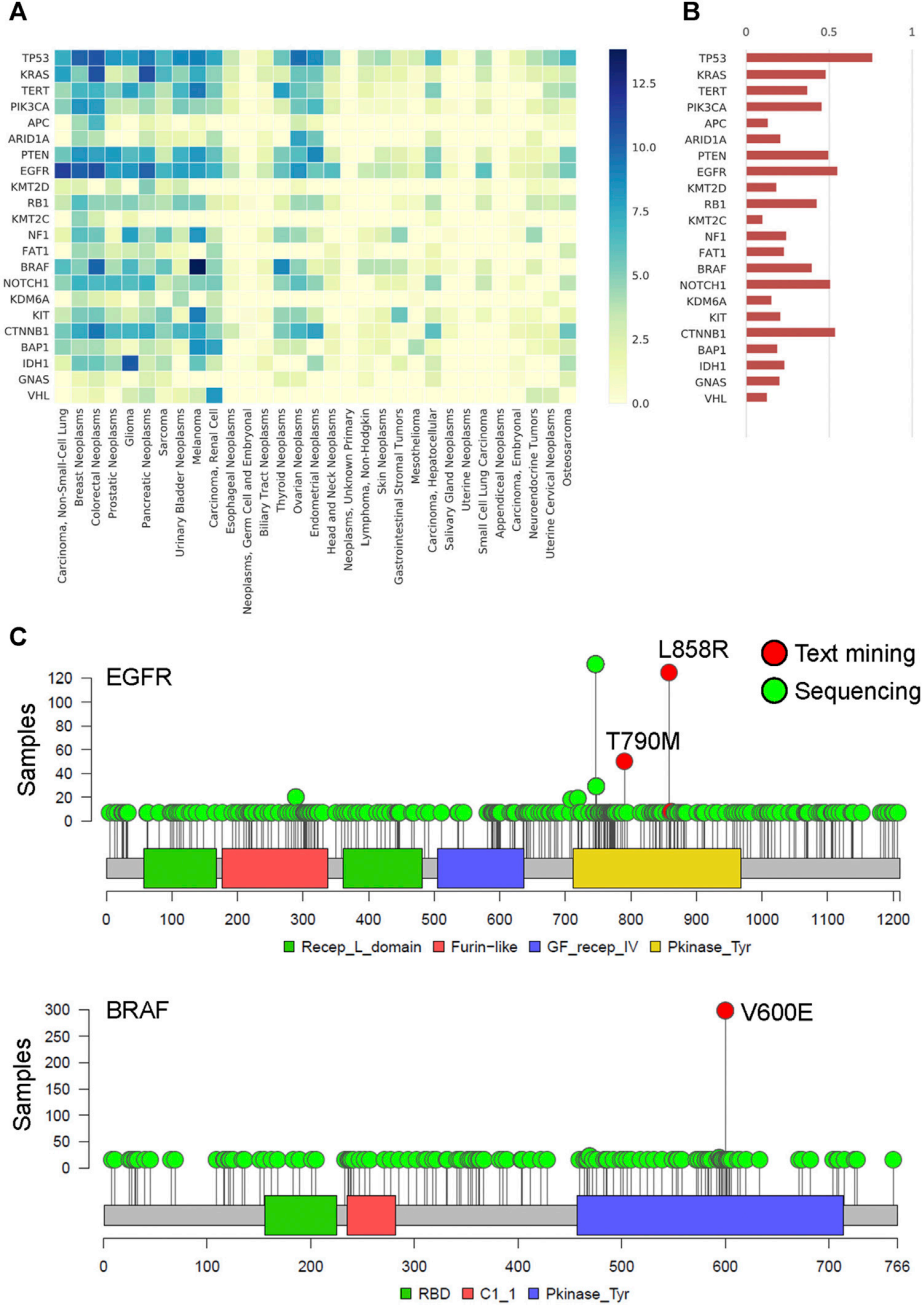
The spectrum and frequency of actionable genetic mutation by literature mining **(A)** Heatmap of cancer genomics by the TF-IDF matrix. The X-axis represents the 31 common cancer types, and the y-axis represents the recurrent somatic genes. The darker color indicates a higher association between genes and cancer. **(B)** The bar plot shows the gene frequency within all of the cancer types. The data is validated by the MSK-IMPACT Clinical Sequencing Cohort, which is targeted sequencing of 10,000 clinical cases using the MSK-IMPACT assay. The cosine similarity of gene frequency between text mining and a statistical result from clinical sequencing data is 80.8%. **(C)** Lollipop plot of *EGFR* and *BRAF* in the MSK-IMPACT pan-cancer cohort. The critical gene mutation term features found by text mining are shown and labeled in red. Other gene mutations are labeled in green.

We constructed the gene-cancer association matrix from the filtered gene term -feature matrix to understand the associations between cancer types and gene mutations. The recurrent common cancer-associated genes are shown in Figure 5A. The most common cancer-associated genes were *TP53*, *EGFR*, *CTNNB1*, *NOTCH1*, and *PTEN*, as shown in Figure 5B. Using two genes, *EGFR* and *BRAF*, as examples, we found that *EGFR* L858R and T790M and *BRAF* V600E were important mutation term features in text mining and were frequently mutated in MSK samples (Figure 5C). The cosine similarity of gene frequency between text mining and a statistical result from clinical sequencing data (Demeester et al., 2016) is 80.8% (Figure 5B). To understand the time series of the association between gene mutations and cancer types in the last decade, we constructed the gene-cancer TF-IDF matrixes of the years from 2011 to 2015 and the years from 2016 to 2019. As shown in Supplementary Figure S2A and S2B, we found that cancer immunotherapy was a major issue in the past 5 years. The rank of CD274 was increased, and CTLA4 first appeared (Seidel et al., 2018). In addition, the TF-IDF value of *BRAF* mutation in colorectal cancers increased because of the better outcomes of the *BRAF*-mutant CRC tumors with microsatellite instability (MSI) in immunotherapy (Rosenbaum et al., 2016). The results indicate that we can design a series of cancer gene panels by updating the literature mining time frame.

### Gene Panel Prediction by Machine Learning Models

**FIGURE 6:**
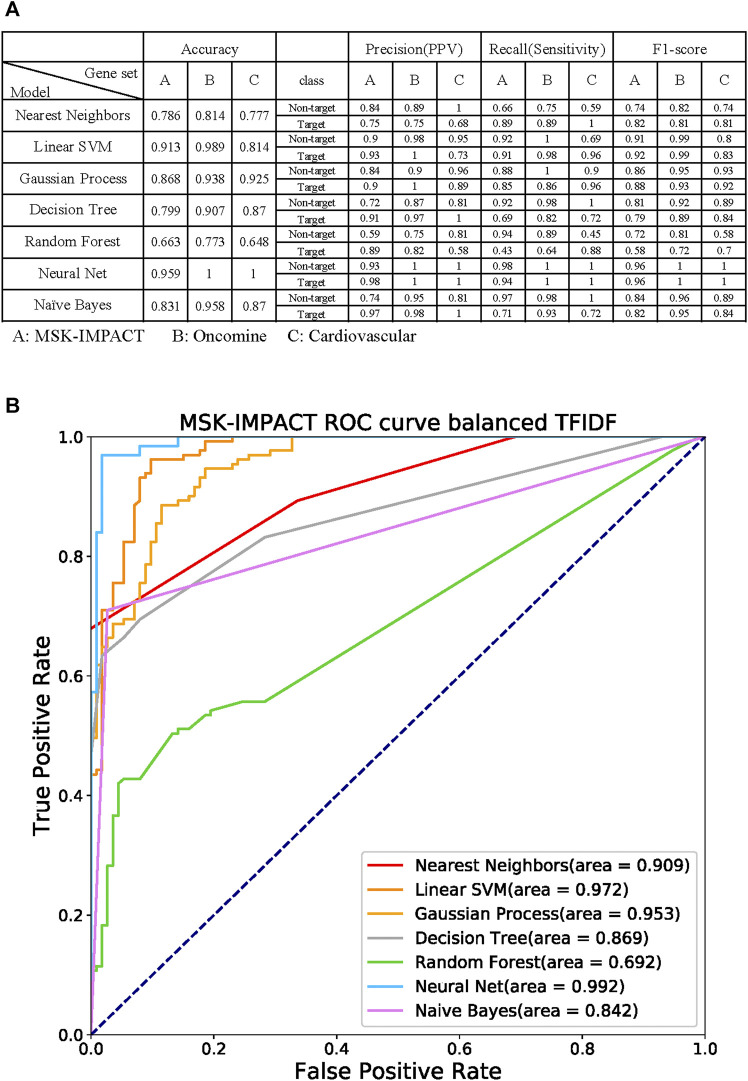
Performance of the machine learning models with the gene panel **(A)** Evaluation of the overall accuracy, precision (positive predictive value, PPV), recall (sensitivity), and F1-score of every prediction model. Each gene could be labeled a target or non-target, indicating whether the gene is in the given target panel. The following seven prediction models were used: nearest neighbors, linear support vector machine (SVM), Gaussian process, decision tree, random forest, neural net, and Naive Bayes. The target gene panels were MSK-IMPACT, Oncomine Comprehensive Assay, and cardiovascular gene panels. **(B)** Receiver operating characteristic (ROC) curves of the models with the MSK-IMPACT 410-cancer gene panel. The neural net model had the highest area under the ROC curve (AUC), which was 0.992.

Seven machine learning prediction models, including nearest neighbors, linear support vector machine (SVM), Gaussian process, decision tree, random forest, neural net, and Naive Bayes (Wei et al., 2015), were used to verify the specific gene panel (Figure 6A). The MSK-IMPACT, Oncomine Comprehensive Assay (Rhodes et al., 2007), and cardiovascular gene panels (Paige et al., 2018) represent different gene characteristics. There are 410 essential cancer genes in the MSK-IMPACT panel. The Oncomine Comprehensive Assay includes 161 cancer-related genes. We used the congenital heart defect focus panel of 115 genes associated with congenital heart defects (CHDs) as the cardiovascular gene panels.

Each gene can be labeled as a target or non-target, which indicates whether the gene is in the given target panel. We performed five-fold cross-validation on our dataset to evaluate the models’ efficiency and evaluate the overall accuracy of each prediction model. We measured the target and non-target genes in each prediction model separately with precision (positive predictive value, PPV), recall (sensitivity), and F1-score. The accuracies for nearest neighbors, linear SVM, Gaussian process, decision tree, random forest, neural net, and naive Bayes in the MSK-IMPACT panel were 0.786, 0.913, 0.868, 0.799, 0.663, 0.959, and 0.831, respectively; the accuracies for all models in the OCP gene panel were 0.814, 0.989, 0.938, 0.907, 0.773, 1 and 0.958; and the accuracies for all the models in the cardiovascular gene panel were 0.777, 0.814, 0.925, 0.87, 0.648, 1, and 0.87. The receiver operating characteristic (ROC) curve analysis confirmed that the neural net model had a better prediction performance; the area under the ROC curve (AUC) was 0.992 (Figure 6B). The AUCs of nearest neighbors, linear SVM, Gaussian process, decision tree, random forest, and naive Bayes were 0.909, 0.972, 0.953, 0.869, 0.692, and 0.842, respectively. The results of the biomedical term feature set prediction models are good, and the performance can reach up to 0.9. This means that the term feature sets can contain most of the information in the gene panel.

### Design of Cancer-Related Gene Panels Based on Topic Modeling

**FIGURE 7:**
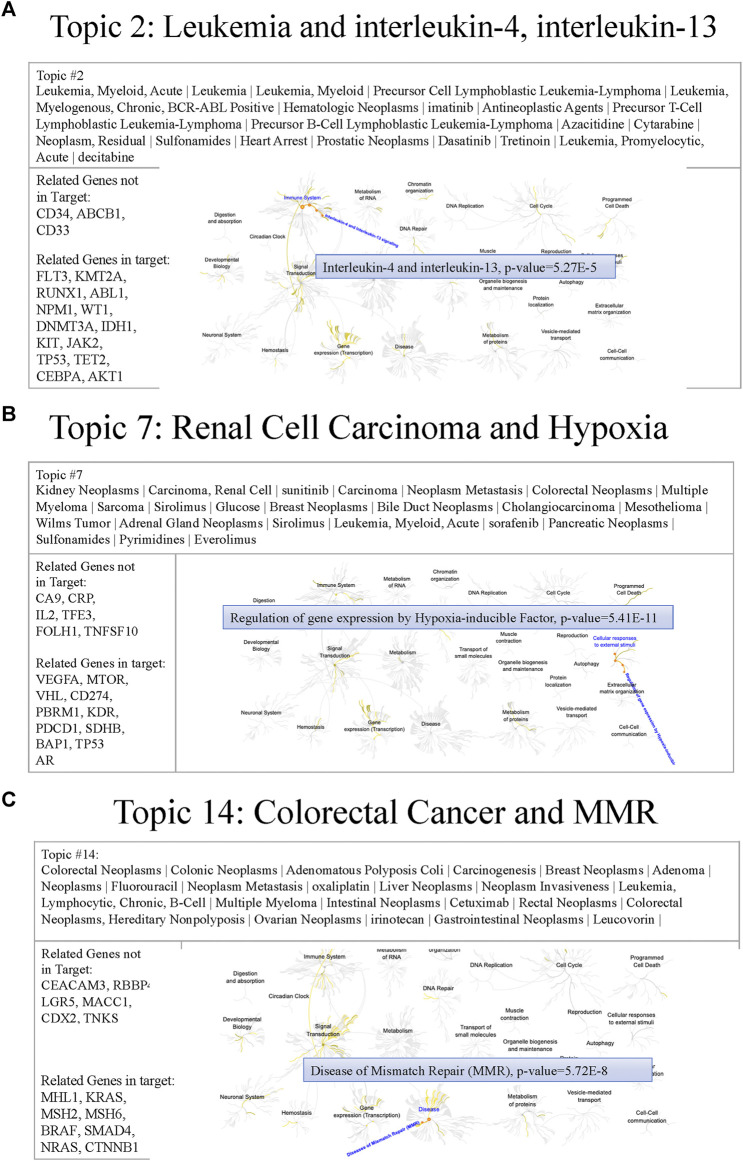
Examples of cancer topics containing relevant text features, genes, and pathways **(A)** Figure showing the text features, genes, and pathways of topic 2. Cancer types (e.g., leukemia) and inhibitors (e.g., imatinib) were reported in this topic. Reactome pathway analysis revealed that the related genes of the MSK-IMPACT panel in topic 2 (e.g., FLT3) were involved in interleukin-4 and interleukin-13 signaling (*p* = 5.27e-5). **(B)** Figure showing the text features, genes, and pathways of topic 7. Text features including cancer types (e.g., kidney neoplasms) and inhibitors (e.g., sorafenib) implied the relationship between topic seven and kidney cancer. The hypoxia pathway enriched by related genes (e.g., VHL) of the MSK-IMPACT panel in topic 7 (*p* = 5.41e-11) played a crucial role in the governance of cancer stem cells of renal cancer. **(C)** Figure showing the text features, genes, and pathways of topic 14. Many text features containing cancer types (e.g., colorectal neoplasms) and inhibitors (e.g., oxaliplatin) indicated the association between topic 14 and colon cancer. Related genes of the MSK-IMPACT panel in topic 14 (e.g., MLH1) were involved in the mismatch repair pathway (*p* = 5.72e-8).

To understand the MSK-IMPACT panel characteristics, we generated thirty topics that potentially represented different biomedical meanings. The following are some examples of issues relevant to genes in the MSK-IMPACT panel. Figure 7 shows the text features, genes, and related pathways derived from the Reactome of topics 2, 7, and 14, including hematologic, and malignancies. In topic two, leukemia subtypes and targeted inhibitors (e.g., imatinib, dasatinib, and decitabine) were mined. Heart arrest, a common side effect of inhibitors for leukemia, was also been reported (Hochhaus et al., 2009). The related MSK-IMPACT panel in topic two was involved in the signaling of interleukin-4 and interleukin-13 (*p* = 5.27e-5), which was associated with the apoptosis of leukemia cells (Chaouchi et al., 1996; Peña-Martínez et al., 2018) (Figure 7A). These results indicated that topic two was associated with leukemia, a hematological malignancy. In topic seven, key text features such as kidney neoplasms, carcinoma, renal cell, and Wilms tumor implied the relationship between topic seven and kidney cancer. Inhibitors for kidney cancer, such as sorafenib and everolimus, were also identified (Martín-Aguilar et al., 2021; Ren et al., 2021). The hypoxia pathway enriched by *VHL*, *VEGFA*, and *PBRM1* (*p* = 5.41e-11) played a crucial role in the governance of cancer stem cells of renal cancer (Myszczyszyn et al., 2015) (Figure 7B). In topic 14, colorectal neoplasms, hereditary nonpolyposis, adenomatous polyposis coli, oxaliplatin, and cetuximab were associated with colon cancer. Related genes (e.g., *MLH1*, *MSH2*, and *MSH6*) in topic 14 were involved in mismatch repair (*p* = 5.72e-8), which has clinical importance in Lynch syndrome (Truninger et al., 2005) (Figure 7C). Other examples of different cancers, including brain cancer, gynecologic cancer, and breast cancer, are shown in Supplementary Figure S3. These results indicated that most of the genes in the MSK-IMPACT panel were collected for either therapeutic usage or biological relevance to various cancer types. In the future, we could design a small subset of multiple-gene groups by cancer topic.

## Disscussion

It is helpful to gain insight into the field that bridges the knowledge gap between valuable biomedical information and free text by text mining (Sachin Kumar Deshmukh, 2020). With biomedical text mining advances and its applications in cancer research, we can design cancer gene panels by the semantic interpretation of comprehensive cancer narratives. Here, we used a biomedical literature mining model to discover the characteristics of a gene panel. Importantly, we demonstrated and validated the performance of the machine learning approach in text mining of cancer information. Our results highlight the following important points. 1) We developed a gene panel analysis framework based on a biomedical text mining pipeline. 2) Our pipeline can enrich the term features of cancer gene panels. 3) We demonstrated and validated the patterns of the cancer mutational landscape by NGS database. 4) The non-negative matrix factorization (NMF) method and topic modeling are useful for generating cancer information. Biomedical literature mining is valuable for discovering the inherent characteristics of gene panels. These results could be applied to the classification of cancer-related information and strategies for novel cancer gene panel designs.

The hypergeometric distribution test is one of the practical machine learning tools in TM. It can be used to select and extract term features from various genomic characterizations (Pal, 2017). We identified the critical term features according to the gene panel using *p*-values based on a hypergeometric test. Our term feature selection methods can distinguish in different gene panels. This implicates a high-performance prediction model for different datasets, including the MSK-IMPACT panel, Oncomine Cancer Panel, and cardiovascular gene panels. Although many gene recommendation algorithms have been developed, little is known about gene panel design.

Our biomedical term tagging algorithm provides a compressive cancer gene panel and related information. With our tagging algorithm, most of the essential biomedical terms in the text have been tagged. The construction of a gene term-feature matrix in different categories provides useful profiling for the characteristics of the genes. In this study, we constructed a biologically meaningful platform to analyze gene panels in terms of the diseases, chemicals, mutations, and MeSH terms related to genes. We can implement more biomedical term feature matrixes, such as a drug-feature matrix and disease-feature matrix. These different types of forms can provide strategies to analyze biology. With NMF topic modeling, we can capture cancer gene-drug information compatible with our knowledge. It will be useful to design a small subset of cancer gene panels by interpreting the topic model.

For the discovery of cancer gene panels, Figure 5A and Figure 7C illustrate an example of a cancer gene panel design for colorectal cancer. The most frequent genes are *KRAS*, *EGFR*, *BRAF*, *PTEN*, *TP53*, *MLH1*, *PIK3CA*, *CTNNB1* in colorectal cancer by the heatmap. Hereditary nonpolyposis colon cancer (HNPCC) is caused by inherited mismatch repair genetic mutations, including *MLH1, MSH2,* and *MSH6.* The lifetime ovarian cancer risk increased in HNPCC. We can find ovarian cancer and a gene panel including *MLH1*, *MSH2*, *MSH6*, *BRAF*, *KRAS*, *SMAD4*, *NRAS*, *CTNNB1* by topic model. In our study, we can design the two different cancer panels by phenotype. These results indicated the platform could provide an opportunity to construct a cancer gene panel recommendation by different cancer subtypes. There are some text mining limitations in our study. The entity-term based features are based only on co-occurrence in three sentences. However, related entities may have distinct relationships, which are not necessarily co-occurred. The features were obtained from only one resource, PubMed abstracts. Many curated databases have many useful biological features of genes or diseases or drugs; for example, Gene Ontology (GO) (Ashburner et al., 2000; The Gene Ontology Consortium., 2017) contains GO terms that describe genes by the functions of genes or cellular components. It may provide a benefit to the cancer researcher. Unfortunately, the TF-IDF table is going to weight toward common diseases and omit those that are critical in identifying rare diseases. The gene panels are not useful for the identification of unknown or rare gene mutations that are important for treatment. Simultaneously, the manuscripts and supplementary materials may also provide more critical results, but the lack of standardization in accessing this information is a significant problem. The text mining method often focuses on a few sentences due to the challenges of creating a complicated relationship between several critical keywords.

As we know, the random forest algorithm performed well than the decision tree in most of pattern classification cases. However, we found that the random forest approach presented a worse ability for cancer gene panel prediction in the experiments. Several reasons may cause this situation in the model training and evaluation, such as whether or not we specify the maximum number of features to be included at each node split. One of the reasons is that the random forest builds subtrees by randomly choosing features from amounts of features in our study. Unlike the other methods, they calculated the weights for each feature by determining the importance of all features. Thus, the performance might be increased when we increase the number of trees in the random forest. Because the subtrees increased, the model will be seen more features to build more diverse trees. Therefore, the model will become robust and make an excellent performance. Nevertheless, in this paper, we are focusing on a pipeline that can contextualize genes. We used the default parameter in most of the methods in our study. Although we are not emphasizing the methods and parameters optimization, it is also an important issue that we will study in our future works.

Several text mining systems have been developed for mutation-disease association (Erdogmus and Sezermen., 2007; Yeniterzi and Sezerman., 2009; Singhal et al., 2016). An automated pipeline using the full-length biomedical literature was recently established and validated by evidence-based gene panels (Saberian et al., 2020). All these methods focus on mutation-disease associations. In contrast, we contextualized the genes for clinical precision medicine. We provide information about druggable targets, mutations in hereditary cancer syndrome, and disease subtypes.

Although many text mining-based gene panel algorithms were developed, there is still little known to validate the gene panel characteristics. This study provides a biomedical literature mining pipeline in gene panel discovery and interpretation. The platform validated by NGS database could provide an opportunity to construct a gene recommendation and annotation system for precision medicine.

## Conclusions

In conclusion, this study highlights the importance of biomedical literature mining in gene panel discovery and interpretation. The platform could provide an opportunity to construct a gene recommendation and annotation system for precision medicine.

## Data Availability

The original contributions presented in the study are included in the article/Supplementary Material, further inquiries can be directed to the corresponding authors.
