# Supplementary figures and images for: Contextualizing Genes by Using Text-Mined Co-Occurrence Features for Cancer Gene Panel Discovery
Source: Front Genet. 2021 Oct 25;12:771435. doi: 10.3389/fgene.2021.771435 (PMC8573063; doi:10.3389/fgene.2021.771435)

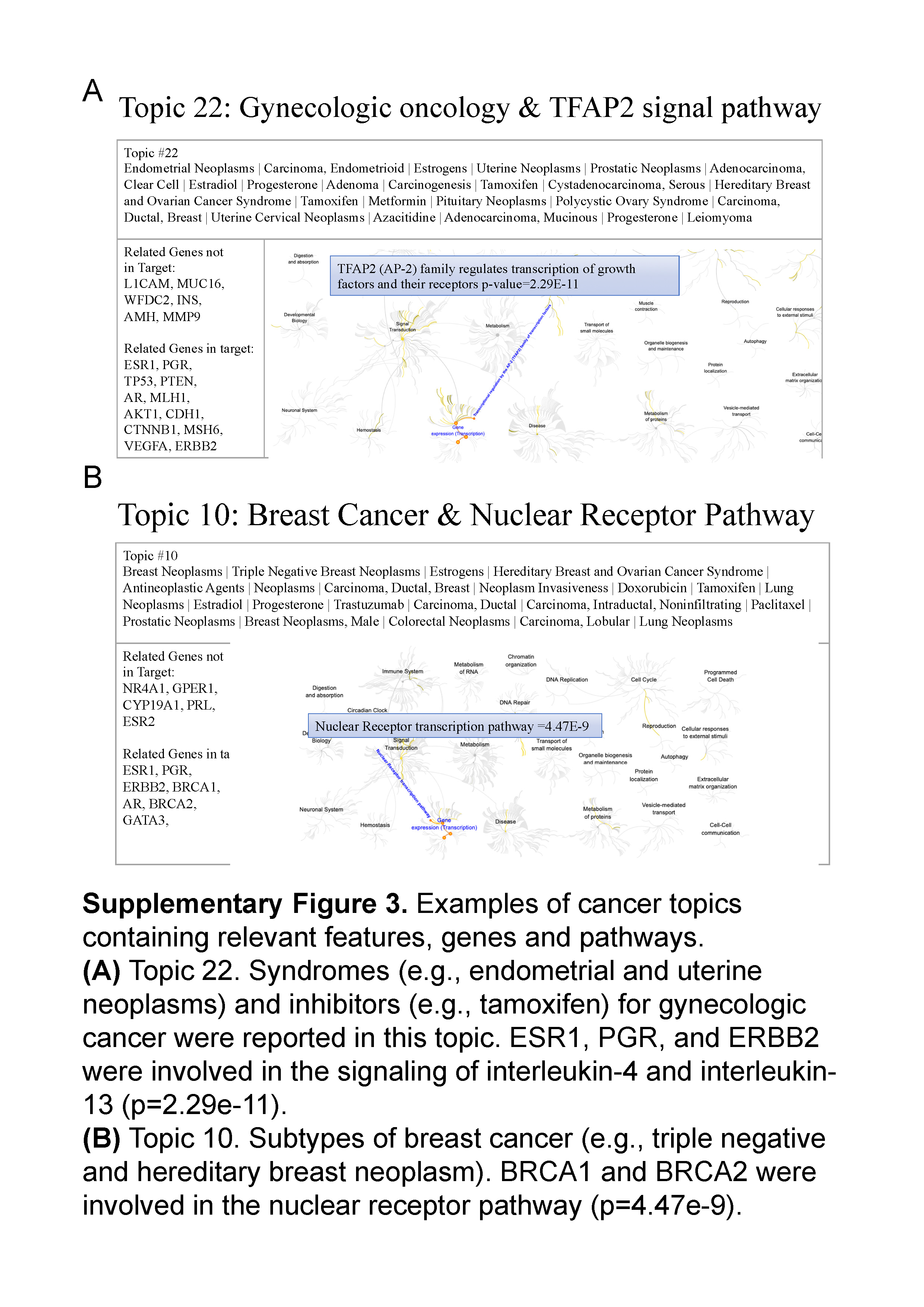

Supplement: Supplementary file 1 [file Image3.TIFF]

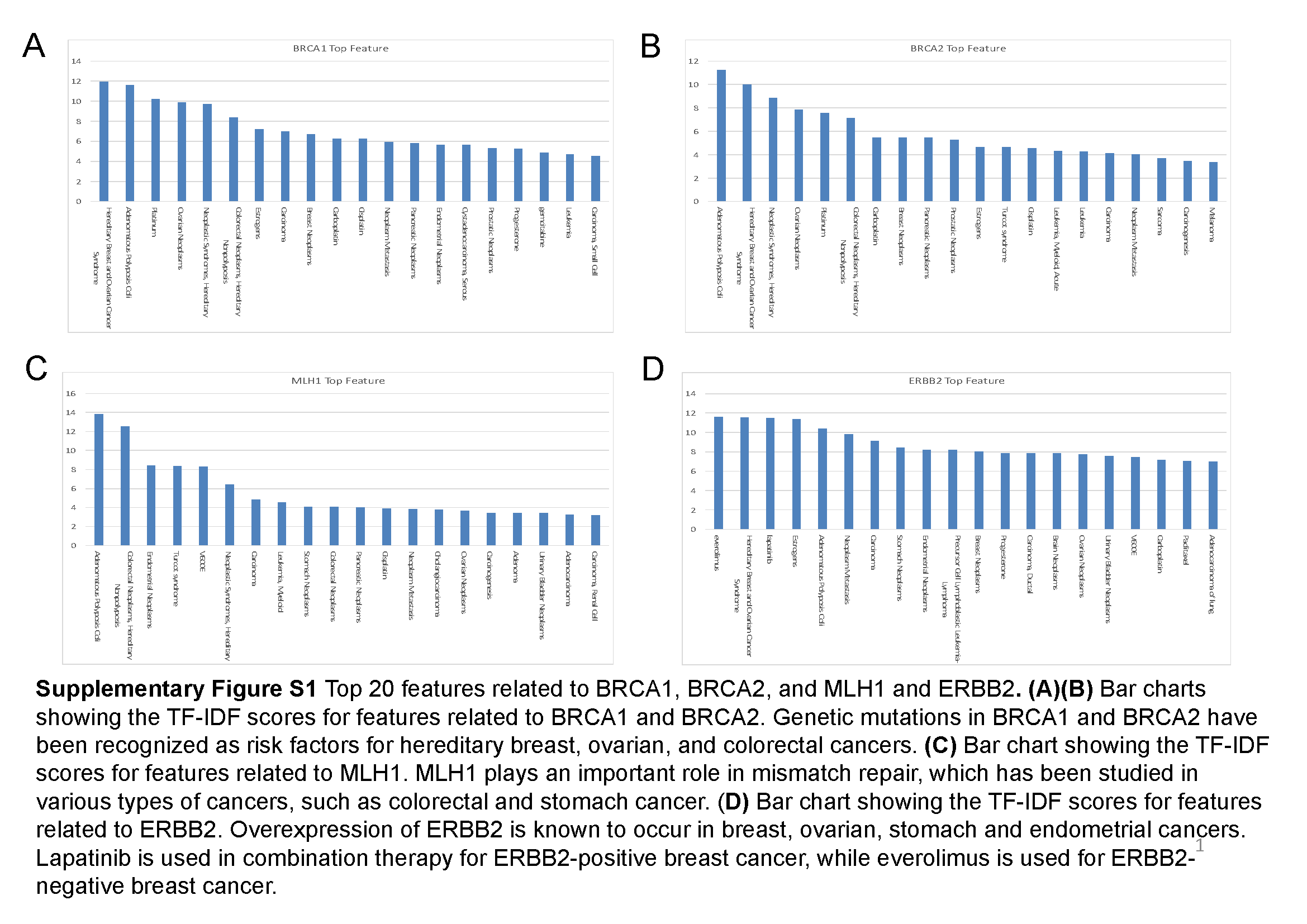

Supplement: Supplementary file 2 [file Image1.TIFF]

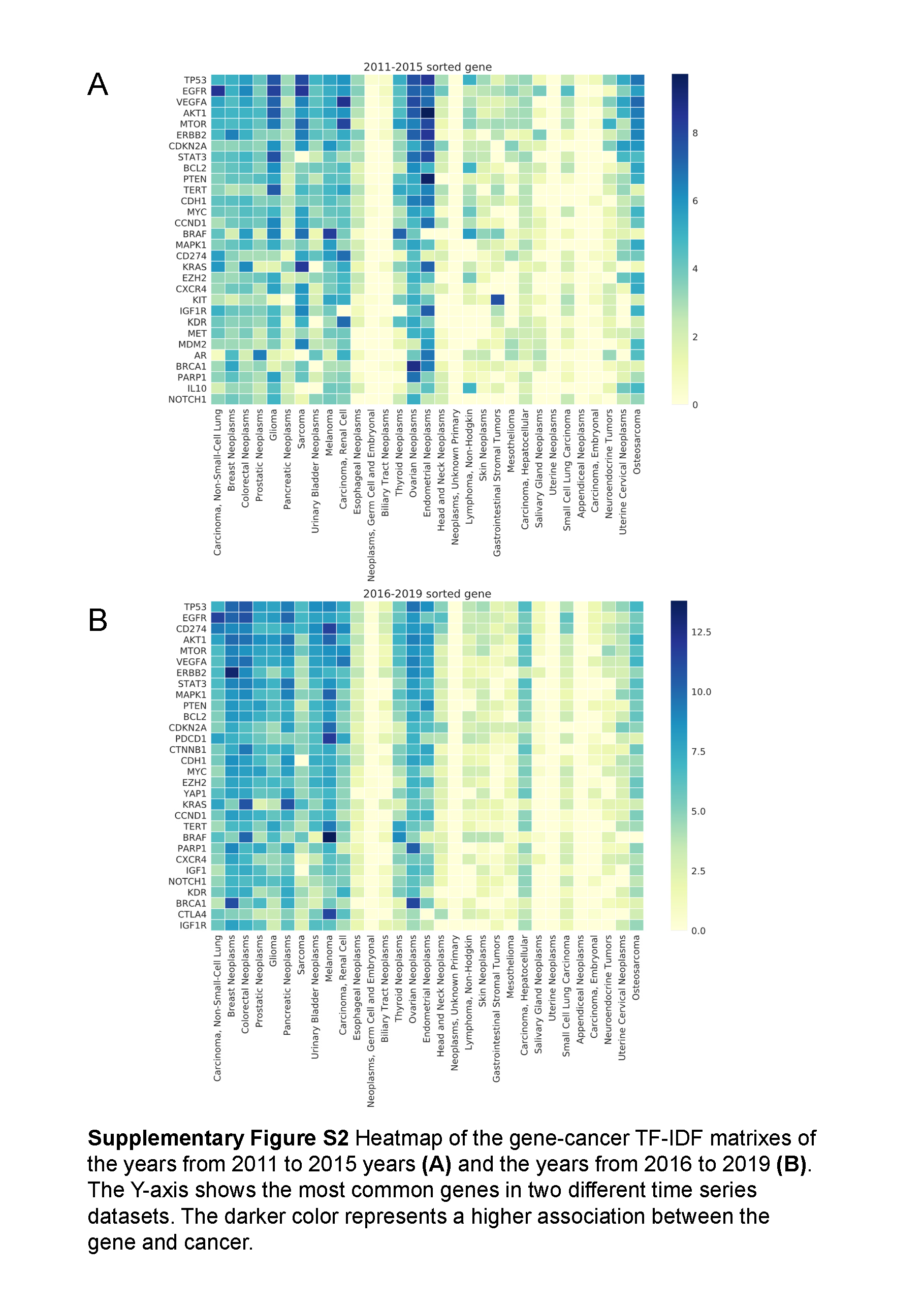

Supplement: Supplementary file 4 [file Image2.TIFF]
